# Cerebellar involvement in warts Hypogammaglobulinemia immunodeficiency myelokathexis patients: neuroimaging and clinical findings

**DOI:** 10.1186/s13023-019-1030-8

**Published:** 2019-02-28

**Authors:** Jessica Galli, Lorenzo Pinelli, Serena Micheletti, Giovanni Palumbo, Lucia Dora Notarangelo, Vassilios Lougaris, Laura Dotta, Elisa Fazzi, Raffaele Badolato

**Affiliations:** 1 Child Neurology and Psychiatry Unit, ASST Spedali Civili Hospital, Brescia, Italy; 2 Clinical and Experimental Sciences Department, University of Brescia, c/o ASST Spedali Civili, 25123 Brescia, Italy; 3 Neuroradiology Unit, Section of Pediatric Neuroradiology, ASST Spedali Civili, Brescia, Italy; 4 Cattedra di Radiologia, University of Brescia, Brescia, Italy; 5 Oncoematologia pediatrica e Trapianto di Midollo, Asst Spedali Civili, Brescia, Italy; 6 Pediatric Unit and “A. Nocivelli” Institute for Molecular Medicine, University of Brescia, ASST Spedali Civili Hospital, Brescia, Italy

**Keywords:** Primary immunodeficiency, CXCR4, Ataxia, Cerebellum, Chemotaxis

## Abstract

**Background:**

Warts Hypogammaglobulinemia Immunodeficiency Myelokathexis (WHIM) syndrome is a primary immunodeficiency characterized by recurrent bacterial infections, severe chronic neutropenia, with lymphopenia, monocytopenia and myelokathexis which is caused by heterozygous gain of functions mutations of the CXC chemokine receptor 4 (CXCR4). WHIM patients display an increased incidence of non-hematopoietic conditions, such as congenital heart disease suggesting that abnormal CXCR4 may put these patients at increased risk of congenital anomalies.

Studies conducted on CXCR4 and SDF-1-deficient mice have demonstrated the role of CXCR4 signaling in neuronal cell migration and brain development. In particular, CXCR4 conditional knockout mice display abnormal cerebellar morphology and poor coordination and balance on motor testing.

**Results:**

In order to evaluate a possible neurological involvement in WHIM syndrome subjects, we performed neurological examination, including International Cooperative Ataxia Rating Scale, cognitive and psychopathological assessment and brain Magnetic Resonance Imaging (MRI) in 6 WHIM patients (age range 8–51 years) with typical gain of functions mutations of CXCR4 (R334X or G336X). In three cases (P3, P5, P6) neurological evaluation revealed fine and global motor coordination disorders, balance disturbances, mild limb ataxia and excessive talkativeness. Brain MRI showed an abnormal orientation of the cerebellar folia involving bilaterally the gracilis and biventer lobules together with the tonsils in four subjects (P3, P4, P5, P6). The neuropsychiatric evaluation showed increased risk of internalizing and/or externalizing problems in four patients (P2, P3, P4, P6).

**Conclusions:**

Taken together, these observations suggest CXCR4 gain of function mutations can be associated with cerebellar malformation, mild neuromotor and psychopathological dysfunction in WHIM patients.

## Background

Warts Hypogammaglobulinemia Immunodeficiency Myelokathexis (WHIM) syndrome is a rare primary immunodeficiency characterized by recurrent bacterial infections, severe congenital neutropenia, with lymphopenia and monocytopenia, associated with myelokathexis in the bone marrow, the latter resulting from the abnormal retention of neutrophils that become senescent [1–4]. Hypogammaglobulinemia is variably present at the onset of the disease. Affected patients display an increased susceptibility to human papillomavirus infection, mainly warts of skin and genitalia, with higher risk of progression to cancer. The recurrence of bacterial pulmonary infections predisposes to chronic lung disease. Congenital heart disease, particularly the tetralogy of Fallot, has been observed in WHIM patients [5]. The syndrome onset is typically early in life with panleukopenia and infections; long-term prognosis correlates to the frequency of infections, and the occurrence of lung disease or malignancy [2]. Non-specific treatments include intravenous or subcutaneous immunoglobulins, Granulocyte Colony-Stimulating Factor (G-CSF) and antibiotic prophylaxis [6]. Targeted therapies are under development and currently represent promising mechanism-based strategies to cure WHIM syndrome [7–9]. All causative autosomal dominant mutations affect the CXC chemokine receptor 4 (CXCR4) and are *gain-of-function* by up-regulating the response to its unique ligand stromal cell derived factor-1 (SDF-1, also called CXCL12) [10, 11]. CXCR4 is a seven-transmembrane G-protein-coupled receptor predominantly expressed by cells of the hematopoietic and central nervous systems [12]. Particularly, well established is the role of SDF1/CXCR4 axis in regulating immune cell homeostasis, trafficking, and chemotaxis [13]. Similarly, studies conducted on CXCR4 and SDF-1-deficient mice have demonstrated the important role of this molecular signaling in neuronal cell migration and brain development [13–16]. In particular, CXCR4 null mice had abnormalities of cerebellar morphology characterized by an irregular external granule cell layer and ectopically located Purkinje cells with poor coordination and balance on motor testing [12–16]. Because WHIM syndrome represents the only Mendelian condition caused by mutation of a chemokine receptor it may provide a human model to understand the role of chemokine signaling not only in immunoregulation but also in embryogenesis and organogenesis. Considering the role of CXCR4 in cerebellum development, the aim of our study was to explore the neuropsychiatric clinical profile together with the possible central nervous system (CNS) involvement, focusing on cerebellar function and structure, in a cohort of WHIM patients.

## Results and discussion

Six female WHIM patients of Caucasian origin (age range 8–51 years) were enrolled for the study. All patients carried heterozygous mutations in *CXCR4* resulting in intracellular truncation of the COOH-terminus: four patients harbored the R334X mutation and two the G336X mutation (Table 1). All the patients, except P1and P2, were previously reported [3]. WHIM syndrome usually becomes manifest in infancy with panleukopenia and recurrent infections. Five patients presented with hypogammaglobulinemia, while myelokathexis was observed in the five patients who underwent bone marrow examination (Table 1).

**Table 1:** Clinical, immunological and genetic data of six patients with WHIM syndrome*

Pt	Age	Sex	Age at onset (yrs)	Mutation (protein)	Warts	Infections	Other	Panleukopenia	Hypogamma globuliemia	Myelokathexis	Current Therapies
P1	9	F	2.5	R334X	Yes (hand)	enteritis, URTI, pneumonia, lymphadenitis	Vitiligo	WBC 740 cells/μl ANC 80 cells/μl AMC 50 cells/ μl ALC 580 cells/μl	Yes IgA 12 mg/dl IgG 529 mg/dl IgM 28 mg/dl	No	Antibiotic prophylaxis, Respiratory FKT (winter time)
P2	12	F	0.5	R334X	No	enteritis,periodontal disease, URTI,sinusitis, recurrent pneumonia	Pulmonary atelectasia	WBC 750 cells/μl ANC 130 cells/μl AMC 70 cells/μl ALC 530 cells/μl	Yes IgA 71 mg/dl IgG 621 mg/dl (on IVIG) IgM 100 mg/dl	Yes	IVIG, Respiratory FKT
P3	18	F	0.3	R334X	Yes (abdomen)	URTI, recurrent pneumonia, enteritis, meningitis	Bronchiectasis, epilepsy	WBC 2390 cells/μl ANC 1790 cells/μl (on G-CSF) AMC 130 cells/μl ALC 430 cells/μl	Yes IgA 22 mg/dl IgG 924 mg/dl (on scIG) IgM 89 mg/dl	Yes	scIG, G-CSF, antibiotic prophylaxis, Respiratory FKT, valproate
P4	21	F	1.9	R334X	Yes (hand, feet)	URTI, otitis, recurrent pneumonia, cellulitis	Tetralogy of Fallot, bronchiectasis	WBC 590 cells/μl ANC 140 cells/μl AMC 70 cells/μl ALC 370 cells/μl	Yes IgA < 7 mg/dl IgG 1110 mg/dl (on IVIG) IgM 99 mg/dl	Yes	IVIG, FKT, antibioitc prophylaxis
P5	32	F	0.3	G336X	Yes (disseminated)	URTI, recurrent pneumonia, otitis	None	WBC 750 cells/μl ANC 390 cells/μl AMC 80 cells/μl ALC 270 cells/μl	Yes IgA 40 mg/dl IgG 250 mg/dl IgM 21 mg/dl	Yes	Antibiotic prophylaxis
P6	51	F	2	G336X	Yes (hand, foot)	pneumonia, parotitis	Melanoma	WBC 940 cells/μl ANC 150 cells/μl AMC 34 cells/μl ALC 697 cells/μl	No IgA 99 mg/dl IgG 810 mg/dl IgM 26 mg/dl	Yes	None

*F* Female, *URTI* Upper Respiratory Tract Infections, *WBC* white blood cells, *ANC* absolute neutrophil count, *AMC* absolute monocyte count, *ALC* Absolute lymphocyte count, *IVIG* Intravenous Immunologbulins, *scIG* subcutaneous immunoglobulins, *FKT* Fisiokinesitherapy. All the lab values were recorded at the time of recruitment. Patients P1 and P2 were not previously reported, while patients P3, P4, P5 and P6 were reported in reference [3] as P6, P1, P2 and P3, respectively

Patients were born at a gestational age of 39.8 ± 0.4 weeks (mean ± SD) (range 39–40 weeks); in all subjects, the prenatal period and birth were uneventful. P3 presented with a mild motor delay with head control, independent sitting and walking reached at the age of 3, 8, and 21 months, respectively, and had been regularly treated with physiotherapy; at the age of 7 years she was diagnosed with developmental coordination disorder. At age of 9 years, she was commenced on valproate for Childhood Absence Epilepsy, and subsequently she was also started with psychotherapy because of obsessive-compulsive disorder associated with anxiety and motor tics. P5 appropriately reached early developmental milestones (head control at the age of 3 months and independent sitting at the age of 6 months), but walking was reached at 21 months; her word production was poor and she underwent speech therapy.

Neurological examination showed signs of mild cerebellar involvement in three patients (P3, P5, P6). In particular, these patients displayed fine and global motor coordination disorders, impaired sequencing of complex motor acts and balance disturbances that resulted in the inability to stand in tandem position in P6, the incapacity to stand on one foot more than 1 s in P3 and P5, and difficulties in manual dexterity and ball skills in all the three patients. These signs were not related to the body weight because it was adequate in all the subjects. Moreover, we observed mild limb ataxia, as all the three girls displayed a segmented movement in the knee-tibia test and an instability in the finger to finger test; In addition, P3 showed a segmented movement in the finger to nose test and slowed and irregular pronation-supination alternating movement. No speech disorders were found, but these three patients presented with excessive but coherent talkativeness. Oculomotor abnormalities were absent in all cases. Patients P3, P5, P6 obtained an International Cooperative Ataxia Rating Scale (ICARS) [17] total score of 6/100, 3/100 and 4/100 respectively, suggesting very mild ataxia. Low ICARS total score (< 7/100) was described in healthy control subjects [18] and in subjects with very mild ataxia [19].

**Fig. 1:**
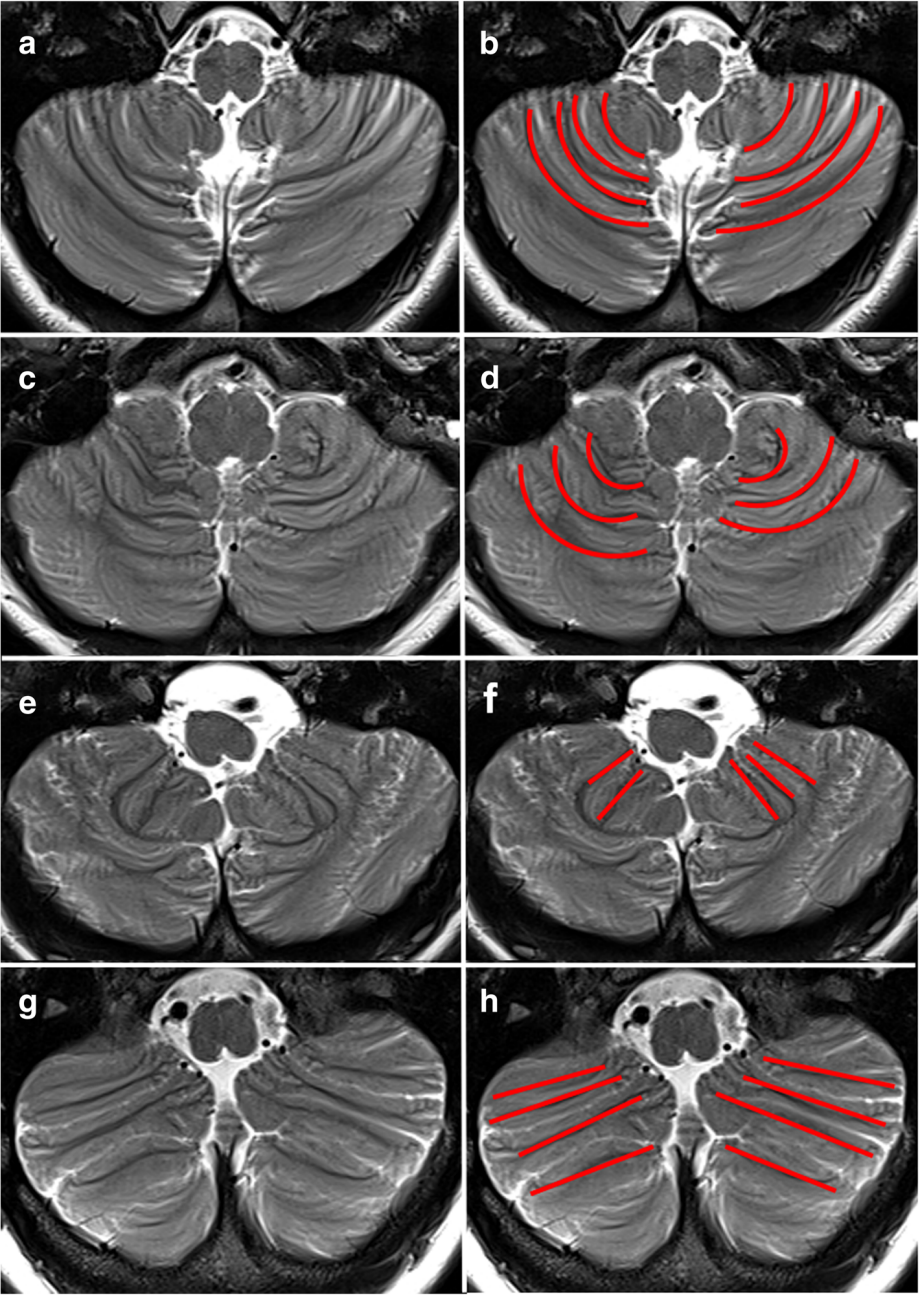
Brain MRI, axial T2-weighted images through the lower cerebellum of a normal subject (**a**, **b**) and three WHIM patients (**c**-**h**). The same MR image is shown in each row: the MR image shown on the right column is presented with an overlying scheme of the foliation pattern (red lines). Panels **a**, **b** Normal subject: the cerebellar folia show a curvilinear course from posteromedial to anterolateral (“onion-like” configuration). Panels **c**, **d**. WHIM patient (P1) with normal “onion-like” orientation of the cerebellar folia. Panels **e**, **f**: WHIM patient (P5): the cerebellar folia show a straight course from posterolateral to anteromedial in a well ordered parallel pattern, confined to anterior portion of the cerebellar hemispheres. Panels **g**, **h**.WHIM patient (P4): the abnormal straight course of the cerebellar folia is more extensive, involving the whole hemispheres

In four patients (P3, P4, P5, P6) brain Magnetic Resonance Imaging (MRI) showed an abnormal orientation of the cerebellar folia confined to the inferior aspect of the hemispheres, involving the gracilis and biventer lobules together with the tonsils (lobules VIIB, VIIIA, VIIIB, IX according to Schmahmann et al. [20] in a bilateral symmetrical fashion. Instead of the normal “onion-like” orientation [21], the folia had a straight course from postero-lateral to antero-medial in a well ordered parallel pattern. This abnormal foliation was more evident in two patients (P3 and P4), where it spanned the whole hemispheres reaching the posterior surface of the cerebellum. This pattern was less evident in P5 and P6, because abnormal folia in the anterior part of the hemispheres coexisted with normal “onion-like” folia in the posterior part of cerebellum (Fig. 1). Cerebellar vermis was considered morphologically normal in all patients. Mean vermis cranio-caudal and antero-posterior diameters in the patients were 4.2 ± 0.26 mm and 2.4 ± 0.22 mm, respectively; mean vermis cranio-caudal and antero-posterior diameters in the control subjects were 4.5 ± 0.24 mm and 2.6 ± 0.25 mm, respectively. The difference of mean cranio-caudal and antero-posterior diameters between patients and control subjects was below two standard deviations, suggesting that vermis size of all patients was within normal range. Three patients had additional cerebral findings in the supratentorial compartment: P3 had a small cystic pineal gland, bilateral hypoplastic olfactory bulbs and unilateral hypoplastic olfactory sulcus; P5 showed an expansible lesion of the pituitary peduncle and deep white matter hyperintensities on T2 weighted-images consistent with gliosis; P6 showed right parieto-occipital and frontal chronic ischemic lesion (due to previous meningitis) and deep white matter hyperintensities on T2-weighted images consistent with gliosis.

The Full Scale Intelligence Quotient (FSIQ) was normal in all patients (97 ± 4.1 standard scores (s.s.); range 90–101). No significant differences were found in Verbal Comprehension (VCI) and Perceptual Reasoning (PRI) (VCI: 102 ± 8.8 s.s.; range 88–110 s.s; PRI: 98 ± 13.7 s.s.; range 80–113 s.s.). The mean Working Memory (WMI) showed a mild decrease if compared to VCI and PRI (93 ± 5,9 s.s.; range 83–100 s.s). Lowest levels were present in the Processing Speed Index (PSI) (91 ± 7,6 s.s.; range 79–100 s.s). P3 and P5 presented a significant difference between VCI and PRI, with a VCI significantly higher than PRI. P1 showed a mild impairment in the task referring to PSI, while P4 showed a mild impairment in WMI. The neuropsychiatric (neurological and cognitive) profile of each patient is summarized in Table 2.

**Table 2:** Neuropsychiatric evaluation (neurological examination, ICARS score and cognitive level)

	Neurological examination	ICARS Score					Intelligence quotient (WISC IV, WAIS IV)				
		Posture & gait	Kinetic	Speech	Oculo motor	Total	VCI	PRI	WMI	PSI	FSIQ
P1	Normal	0/34	0/52	0/8	0/6	0/100	108	113	94	79	101
P2	Normal	0/34	0/52	0/8	0/6	0/100	88	100	91	91	90
P3	Altered	1/34	5/52	0/8	0/6	6/100	109	80	95	87	95
P4	Normal	0/34	0/52	0/8	0/6	0/100	104	102	83	97	100
P5	Altered	1/34	2/52	0/8	0/6	3/100	96	110	97	95	99
P6	Altered	2/34	2/52	0/8	0/6	4/100	110	83	100	100	98

*ICARS* International Cooperative Ataxia Rating Scale, *WISC IV* Wechsler intelligence scales IV edition, *WAIS IV* Wechsler adult intelligent scales IV edition, *VCI* Verbal Comprehension Index, *PRI* Perceptual Reasoning Index, *WMI* Working Memory Index, *PSI* Processing Speed Index, *FSIQ* Full Scale Intelligence Quotient

As regards to psychopathological symptoms, three patients (P2, P3, P6) presented scores above the borderline range cut-points on the scale for internalizing problems, while three (P2, P4, P6) had borderline clinical scores on the scale for externalizing problems. Moreover, we observed affective problems (i.e. anxiety/depression and/or withdrawal) in five patients. In the syndrome scale for anxiety/depression three subjects (P2, P3, P6) presented scores above the borderline range cut-points. Four patients (P3, P4, P5, P6) reached the borderline clinical scores in syndrome scale for withdrawal. P3 and P6 showed the highest scores in the affective scales. Somatic complaints were reported in two patients (P2 and P6), attentive problems were detected in three patients (P2, P3, P5) and aggressive behavior in one case (P2). No patients reported rule breaking behaviors. P2, P3 and P6 reported the greatest number of risk signs of psychopathological symptoms; only P1 showed scores within the normal range. Vineland Adaptive Behavioural Scales II (VABSII) [22] showed mean normal standard scores in domains of Communication, Daily living skills and Socialization in five out of six patients. P3 presented significant difficulties in the organization of daily living and socialization skills. The neuropsychiatric assessment related to psychopathological evaluation is summarized in Table 3.

**Table 3:** Neuropsychiatric evaluation (psychopathological profile and adaptive functions)

	Psychopathological symptoms (CBCL/ ASR) T Scores								Adaptive functions (VABs) Percentiles		
	Int probl	Ext probl	A/D	WIT	SC	AP	AB	RBB	COM	DLS	SOC
P1	48	47	51	50	57	53	52	53	23	45	79
P2	67	65	65	51	79	63	66	58	32	39	39
P3	66	52	68	73	52	66	51	51	75	< 1	< 1
P4	57	63	53	62	58	56	56	56	42	63	66
P5	59	58	58	62	56	66	58	58	66	79	50
P6	69	63	68	65	65	57	59	59	66	68	50

*CBCL* Child Behavior Checklist 6–18, *ASR* Adult Self-Report, *A/D* Anxious/Depressed, *WIT* Withdrawn, *SC* Somatic Complaints, *AP* Attention Problems, *AB* Aggressive Behaviour, *RBB* Rule breaking behavior, *VABs* Vineland Adaptive Behavioural Scales II, *COM* Communication skills, *DLS* Daily living skills, *SOC* Socialization

The present study describes the neuropsychiatric manifestations and CNS anomalies in a cohort of WHIM patients with *gain-of-function* CXCR4 mutations. The role of the CXCR4 gene in embryogenesis and organogenesis has been investigated in murine models of embryogenesis and immune system development. CXCR4 is essential at the earliest stages of B-cell lymphopoiesis, for colonization of bone marrow by multipotential hematopoietic cells, for cardiac septum formation and brain morphogenesis [12–16, 23]. CXCR4 mRNA transcripts are expressed in various regions of the developing brain, including the retina, olfactory epithelium, olfactory bulb, hippocampus, spinal cord and,above all, cerebellum [15]. It is known that knock-out mice do not survive the fetal stage, while CXCR4 null mice exhibit impaired cerebellar folia development, lower density of differentiating granule cells and abnormal migration of Purkinje cells. From a clinical point of view, CXCR4 null mice performed poorly in coordination, muscle strength, balance, and skilled walking tests [12]. As observed in animal studies, our study has revealed clinical and morphological signs of cerebellar involvement in a significant proportion of WHIM patients. Half of the patients that we studied presented with mild cerebellar symptoms characterized by fine and global motor coordination disorder, impaired sequencing of complex motor acts, balance impairment and limb ataxia, as revealed by an accurate neurological examination and ICARS, while the other three were normal. All patients showed a normal Intelligence Quotient, but Working Memory and Processing Speed Indexes were slightly decreased in half of them. Verbal skills were significantly better than visual perceptual skills in two patients, who also presented with excessive but coherent talkativeness. Increased risk of internalizing and/or externalizing problems were observed in our cohort as evaluated by Child Behavior Checklist 6–18 (CBCL) and by the Adult version of the test (Adult Self-Report -ASR-) [24, 25]; in particular, affective problems were detected. Adaptive skills were within normal range in most of the patients. The neurological symptoms characterized by coordination disorder, balance impairment and limb ataxia, the presence of greater verbal performance on intellectual assessment and the significant risk of psychopathological disorders have been previously described in subjects with cerebellar dysfunction [26, 27]. Cerebellum has been associated with coordination movements and control of posture [26] but more recent studies have provided evidence of its involvement in higher cognitive and emotional functions [27]. Schmahmann and Sherman used the term “Cerebellar-Cognitive Affective Syndrome (CCAS)” to describe the specific neuropsychological profile observed in subjects with cerebellar lesions [28]. In detail, CCAS is characterized by an impairment of executive functions, visuo-spatial abilities, speech and language and by personality change [26–28] related to the abnormalities of the white matter tracts connecting the cerebellum with brainstem and cerebral hemispheres [27–29]. Nevertheless, the higher risk of psychopathological problems in our cohort may be related to the effect of a chronic condition, such as WHIM syndrome represents, on behavioral competence [30]. Subjects with chronic diseases (such as chronic idiopathic urticaria...) exhibited a higher risk of depression, trait anxiety, internalizing problems, somatic complaints in the CBCL when compared with healthy controls [29]. Daily stressors involved in disease management might increase psychological distress and contribute to psychiatric comorbidity [30, 31]. Instead a chronic disease (such as juvenile rheumatoid arthritis) seems not to have a negative impact on VABS daily living skills and VABS socialization [32].

CXCR4 has been demonstrated to be involved in cerebellar morphogenesis [15]. Brain MRI can accurately depict cerebellar morphology and the cerebellar folia. On axial sections of each hemisphere MRI can reveal the typical “onion-like” pattern, with curved shape parallel to the occipital squama, running from posteromedial to anterolateral. On the contrary, in four WHIM patients, cerebellar folia of the lower hemisphere were running in a straight course, parallel to each other, from posterolateral to anteromedial (Fig. 1). The cerebellar foliation abnormality had different degrees of severity in these four patients; in two of them the abnormal foliation spanned the whole lower hemisphere, while in the other two this anomaly was confined to its most anterior part. However, the number of WHIM patients that we have been able to study was too small to draw a strong correlation between the neuroradiological signs and the clinical findings. Among WHIM patients three of them presented the cerebellar foliation abnormality, motor coordination disorders, impaired sequencing of complex motor acts, balance disturbances and mild limb ataxia, suggesting a correlation of the neurological symptoms with the neuroradiological findings. In contrast, patient P4 who presented the typical cerebellar anomaly did not show balance impairment or other coordination disorders. In addition, we did not observe any correlation of the neurological and cerebellar imaging anomalies with CXCR4 genotype because they were observed in patients with R334X or G336X mutation. Therefore, we cannot draw final conclusions about any genotype phenotype correlation, and the clinical significance of the cerebellar foliation pattern abnormality that we have observed, suggesting that these studies need to be extended to a larger number of patients with WHIM syndrome.

In the last years, several cerebellar disorders characterized by abnormal foliation, collectively named cortical cerebellar dysplasia, have been described in sporadic cases with unknown cause [33] or in the setting of specific syndromes [34–36] Some of these disorders typically associate cerebellar cysts (Poretti-Boltshauser syndrome due to LAMA1 mutations [35], alphadystroglycanopathies [34], GPR56 mutations [37], COL3A1 mutations [38]), others show only abnormal foliation (Chudley McCullough syndrome due to GPSM2 mutations [39], or the so-called “tubulinopathies” due to mutations in tubulin genes [40]). To our knowledge, in all these disorders cerebellar abnormality consists of an irregular, disorganized and often asymmetric foliation, different from the well-ordered (yet abnormal) pattern of foliation we found in WHIM patients.

## Conclusions

We conclude that CXCR4 *gain-of-function* mutations may be associated with a mild cerebellar malformation and mild neuromotor and neuropsychological dysfunctions. These findings could be explained by the altered CXCR4-CXCRL2 signaling that in WHIM syndrome might cause the abnormal migration of Purkinje cells similarly to the abnormal trafficking that has been largely demonstrated for hematopoietic cells. A limitation of this study is the reduced number of subjects recruited due to the rarity of the disease that prevents the possibility of any statistical significance.

Based on our results, a complete neuropsychiatric evaluation and brain MRI should be performed in all WHIM patients in order to demonstrate any morphological abnormality of the cerebellum and provide additional information on their global functioning, in order to help clinicians in the management of affected patients. Neuroradiologists should be aware of the distinct cerebellar foliation pattern that can be associated with WHIM syndrome. In a patient without an established diagnosis, undergoing brain MRI because of cerebellar issues, the peculiar cerebellar foliation abnormality could be a hint to the diagnosis of WHIM syndrome.

## Methods

### Patients

Six patients with the diagnosis of WHIM syndrome regularly followed at the Department of Pediatric of the ASST Spedali Civili of Brescia were included in this study. All patients are Italian females with an age of 22.5 ± 15 years (age range 8–51 years) and were recruited between November 2016 and July 2017. The study was conducted in accordance with the ethical guidelines set forth by the Declaration of Helsinki and was approved by the Ethical Committee of the ASST Spedali Civili of Brescia, Italy. Written informed consent was obtained from all participants and/or their parents, if minors. Clinical and laboratory data were collected from medical records.

### Assessments

All patients were deeply investigated in their family and past medical history. They underwent a comprehensive neuropsychiatric evaluation that included the neurological examination and assessment of cognitive, psychopathological profile and adaptive functions. All the tests and the neurological examination were performed once.

The neurological examination focused specifically on cerebellar signs and symptoms. The International Cooperative Ataxia Rating Scale (ICARS), a scale of cerebellar ataxia symptoms [41] was applied. ICARS is a 100-point semi-quantitative scale comprising 19 items grouped into 4 subscales: Posture and Gait disturbances (maximum score = 34), Kinetic Functions (maximum score = 52), Speech Disorders (maximum score = 8) and Oculomotor Disorders (maximum score = 6). Scores for each subscale quantify the extent of ataxia in each clinical area such as higher scores represent greater severity of the ataxia. Subscale scores are summed to give a total score ranging from 0 (reflecting no ataxia) to 100 (representing maximum ataxia). Cognitive level was evaluated using age-appropriate versions of Wechsler intelligence scales IV edition [42] and Wechsler adult intelligent scales IV edition [43]. Full Scale Intelligence Quotient (FSIQ), Verbal Comprehension (VCI), Perceptual Reasoning (PRI), Working Memory (WMI), and Processing Speed Index (PSI) scores were collected. All the quotients are reported in standard scores (mean 100, SD 15).

The psychopathologic profile was explored according to patients’ age: Child Behavior Checklist 6–18 was administered to parents if minors [24], while the Adult version of the test was proposed to adult patients [25]. The Italian versions of CBCL and ASR scales were obtained through back-translation authorized and approved by T. Achenbach. The checklist provides series of questions that assess behavioral competency and behavioral problems within the past six months, using 0 as “never”, 1 as “sometimes present” and 2 as “always present”. The total value attributed to each item gives a score which is converted into a T score and whose value expresses the severity of the problem for the patient studied. The cut off points for total problems were calculated using T scores and categorized thus: up to 60 points as normal; between 61 and 69 points as borderline, and from 70 points as clinical for a particular problem. Anxious/Depressed, Withdrawn, Somatic Complaints, Attention Problems, Aggressive Behavior and Rule-Breaking Behavior were considered in this study. Another level of classification aggregates the sub-groups Withdrawn, Somatic Complaints, Anxious/Depressed into a scale evaluating problems designated ‘internalization’. The ‘externalization’ evaluation scale based on the following sub-groups: Aggressive Behavior and Rule breaking behavior. Cut-off points of clinical interest were identified according to the tool kit software standards. Adaptive functions were evaluated by proponing to patients’ parents or relatives the Vineland Adaptive Behavioural Scales II [22]. VABS II is composed by three domains: Communication (subdomains: Receptive, Expressive and Written Communication), Daily living skills (subdomains: Domestic, Personal and Community Skills) and Socialization (subdomains: Interpersonal Relationships, Play/Leisure, Coping Skills). Scores of each domain were expressed in percentiles. Adaptive disorders were considered for those domains which obtained a centile score under 5.

All patients underwent brain MRI to assess brain morphology. The first patient examined was studied because of clinical indication (i.e. epilepsy and developmental coordination disorder) with 1.5 Tesla MR scanner (Magnetom Aera; Siemens Healthcare, Erlangen, Germany) with a 20 channel head coil. The MR protocol included 3D-T1-weighted Magnetization-Prepared Rapid Gradient-Echo (MPRAGE) sections (TR/TE = 2040/3.08 ms; slice thickness = 0.83 mm; matrix = 320 × 320), T2-weighted Turbo-Spin-Echo (TSE) axial sections (TR/TE = 4960/116 ms; slice thickness = 5 mm, matrix = 512 × 358), T2-weighted TSE coronal sections (TR/TE = 5080/114 ms; slice thickness = 3 mm, matrix = 384 × 278), T2 Fluid Attenuated Inversion Recovery (FLAIR) axial sections (TR/TE = 8800/99; slice thickness = 5 mm, matrix = 320 × 218), T1-weighted Spin-Echo post-contrast axial sections (TR/TE = 663/17; slice thickness = 5 mm, matrix = 320 × 227). The next five patients were studied prospectively with a 3 Tesla MR scanner (Magnetom Skyra; Siemens Healthcare, Erlangen, Germany) with a 64 channel head coil and a dedicated imaging protocol structured as follows: 3D-T1-weighted MPRAGE sections (TR/TE = 2300/2.43 ms; slice thickness 0.83 mm; matrix = 320 × 320), T2-weighted TSE multiplanar sections (TR/TE = 4030/128 ms; slice thickness 3 mm, matrix = 512 × 358), T2-weighted FLAIR axial sections (TR/TE = 9000/92 ms; slice thickness 3 mm, matrix = 320 × 240). A neuroradiologist with more than 20 years of experience evaluated the MR images, focusing on cerebral cortex, hippocampus and cerebellum, given the role of CXCR4 in the development of these structures; regarding cerebellum, foliation, fissuration and cortical thickness were qualitatively evaluated and compared to normal subjects. Vermis size (cranio-caudal and antero-posterior diameters) was also assessed by comparison with sex−/age-matched normal subjects (two controls each patient) due to absence of normative data on cerebellar vermis size for post-natal MRI. Normal controls were recruited among patients undergoing brain MRI for reason unrelated to cerebellar issues (i.e., primary headache, sinonasal disease, positive family history for brain aneurysm) with normal neurologic examination.
